# Anaplastic Lymphoma Kinase Acts in the *Drosophila* Mushroom Body to Negatively Regulate Sleep

**DOI:** 10.1371/journal.pgen.1005611

**Published:** 2015-11-04

**Authors:** Lei Bai, Amita Sehgal

**Affiliations:** 1 Howard Hughes Medical Institute, University of Pennsylvania, Philadelphia, Pennsylvania, United States of America; 2 Department of Neuroscience, Perelman School of Medicine, University of Pennsylvania, Philadelphia, Pennsylvania, United States of America; Washington University Medical School, UNITED STATES

## Abstract

Though evidence is mounting that a major function of sleep is to maintain brain plasticity and consolidate memory, little is known about the molecular pathways by which learning and sleep processes intercept. Anaplastic lymphoma kinase (*Alk*), the gene encoding a tyrosine receptor kinase whose inadvertent activation is the cause of many cancers, is implicated in synapse formation and cognitive functions. In particular, *Alk* genetically interacts with *Neurofibromatosis 1* (*Nf1*) to regulate growth and associative learning in flies. We show that *Alk* mutants have increased sleep. Using a targeted RNAi screen we localized the negative effects of *Alk* on sleep to the mushroom body, a structure important for both sleep and memory. We also report that mutations in *Nf1* produce a sexually dimorphic short sleep phenotype, and suppress the long sleep phenotype of *Alk*. Thus *Alk* and *Nf1* interact in both learning and sleep regulation, highlighting a common pathway in these two processes.

## Author Summary

Animal and human studies suggest that sleep has a profound impact on learning and memory. However, little is known about the molecular pathways linking these phenomena. We report that mutations in the *Drosophila* Anaplastic lymphoma kinase (*Alk*) gene, an ortholog of a human oncogene ALK, cause increased sleep. ALK is required for sleep suppression in the mushroom body, a structure important for both sleep and memory. ALK generally activates the Ras/ERK pathway, which is negatively regulated by Neurofibromin 1 (NF1). Mutations in *Nf1* are the causes of the common neurological disorder Neurofibromatosis type 1 (NF1), which affects 1 in 3,000 live births. We find that male flies lacking the NF1 protein have reduced sleep, a phenotype opposite that of *Alk* flies. Interestingly, even though mutations in *Nf1* don’t always cause short sleep in female flies, they suppress the sleep increase induced by ALK inactivation. Previous studies have shown that *Alk* and *Nf1* play antagonistic roles in learning and that both genes regulate synaptic growth. Thus *Alk* and *Nf1* interact to regulate both sleep and learning, suggesting that the two processes share a common pathway. Our results support a model in which changes in synaptic plasticity during sleep promote learning and memory.

## Introduction

Sleep behavior is conserved from worms and insects to fish and mammals [1]. Why animals spend a large amount of time seemingly doing nothing, passing on opportunities to forage, hunt or mate, and remaining vulnerable to dangers, is still a mystery. It is hypothesized that a major function of sleep is to maintain brain function, in particular, to ensure synaptic homeostasis of neurons and to consolidate memory [2,3]. Levels of synaptic proteins are associated with sleep/wake states in both flies and mammals [4,5], and sleep deprivation impairs memory formation in a variety of species, including humans [6], mice [7], and *Drosophila* [8,9]. In addition, some molecules that regulate learning and memory turn out to be required for sleep/wake regulation [10]. However, only a handful of such molecules have been identified and for most it is not known if effects on the two processes are mechanistically linked.

*Anaplastic lymphoma kinase* (*Alk*), which encodes a member of the ALK/LTK (leucocyte tyrosine kinase)) family of receptor tyrosine kinases (RTKs), is proposed to play important roles in the nervous system based on its extensive expression in the CNS of both mammals and flies [11–14]. Its *in vivo* functions are mostly studied in the context of *Drosophila* development. Together with its secreted ligand Jelly Belly (Jeb), ALK is essential for 1) gut muscle differentiation [15,16]; 2) retinal axon targeting in the optic lobe [17]; 3) growth and organ size regulation [14,18]; and 4) modulation of neuromuscular transmission and synapse growth at larval neuromuscular junctions (NMJ) [19]. However, there is also evidence for a role of *Alk* in brain plasticity in adult contexts. Adult-specific activation of *Alk* causes deficits in associative olfactory learning in *Drosophila*; concordantly, reducing neuronal *Alk* activity in adult flies enhances olfactory learning [14]. Similarly in mice, loss of *ALK* function enhances spatial memory and novel object recognition, and reduces anxiety and depression [20,21]. Effects of *Alk* on learning, at least in flies, are most likely mediated by Ras/ERK signaling. This is supported by an interaction with *Nf1*, conserved ortholog of the human Neurofibromatosis type 1 (NF1) disease gene, which encodes a GTPase-activating protein (GAP) that negatively regulates Ras/ERK signaling. Specifically, the learning deficit in *Nf1* flies is rescued by down regulation of *Alk* [14].

Based on the role of *Alk* in neuronal plasticity and learning, we hypothesized that *Alk* may be involved in sleep regulation. We found that inactivation of ALK causes increased sleep. We probed for the neuronal circuit that underlies *Alk*’s involvement in sleep and found that inhibiting *Alk* in the mushroom body induces more sleep, suggesting *Alk* as a mechanistic link between learning and sleep. In addition, *Alk* interacts with *Nf1* in the regulation of sleep just as it does in the context of learning. *Nf1* also have circadian phenotypes [22], but *Alk* is not required for circadian rhythms, nor does it interact with *Nf1* in the circadian regulation of rest/activity rhythms. Thus, interactions between the two molecules are specific for sleep and learning.

## Results

### *Alk* mutants have increased sleep

Because ALK plays a crucial role in gut development, the null allele *Alk* ^*1*^ is homozygous lethal at the early larval stage [23]. However, a temperature-sensitive allele, *Alk* ^*ts*^, fully complements *Alk* ^*1*^ at 18°C, but fails to complement developmental *Alk* ^*1*^ lethality at 29°C [19]. We were therefore able to raise *Alk* ^*ts/1*^ trans-heterozygous or *Alk* ^*ts*^ homozygous flies to the adult stage at 18°C and prevent developmental phenotypes such as changes in body length (S1 Fig). *Alk* mutants raised in this manner are presumably also spared other developmental defects seen with manipulations of ALK activity, such as altered NMJ structure and function [19] and mis-targeting of retinal neurons in the optic lobes [17].

**Fig 1:**
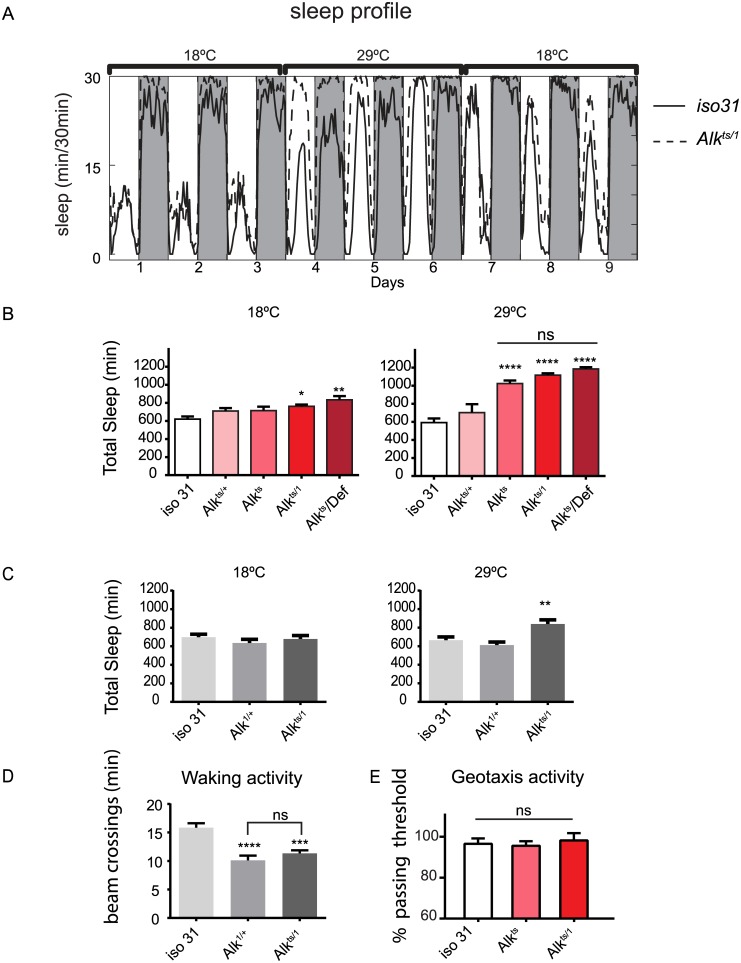
*Alk* mutants have increased sleep. A) The restrictive temperature of 29°C reversibly increases sleep in *Alk* ^*ts/1*^ mutants. The averaged sleep profiles, plotted as average amounts of sleep in every 30-minute period, are shown for *iso31* and *Alk* ^*ts/1*^ female flies. The white and the grey columns mark periods of day and night, respectively. N = 16. B) Quantification of average daily sleep of control flies and *Alk* mutants measured by single beam monitors. Total sleep at 18°C was calculated as the average of 3 days before the temperature shift to 29°C. Total sleep amounts at 29°C were calculated as the average of the 3 days following the temperature shift and are significantly different between genotypes (One-way ANOVA, p<0.0001). Asterisk* signifies difference from *iso31* control. In this figure and all following figures, *, p<0.05; **,p<0.01; ****,p<0.0001; ns, not significantly different. Error bars are SEM. N = 13–16. C) Measurement by multi-beam monitors similarly revealed longer sleep in *Alk* ^*ts/1*^ flies at the restrictive temperature. There is no difference between genotypes at 18°C (p = 0.4722), while at 29°C *Alk* ^*ts/1*^ sleep significantly longer than *iso31* and *Alk* ^*1/+*^ flies. N = 15–16. D) Waking activity in the multi-beam monitors was measured at the restrictive temperature of 29°C and was defined as averaged number of beam crossings per minute during wake. N = 15–16. E) Normal negative geotaxis response in *Alk* mutants. The negative geotaxis response is measured as the percentage of flies climbing vertically >4 cm from the bottom of a vial within 10s of being tapped down. N = 5 (groups of 10 flies for each genotype).

We assayed sleep using the traditional single infrared beam interruption device (Trikinetics, MA) in adult *Alk* ^*ts/1*^ and *Alk* ^*ts*^ female flies at the permissive temperature of 18°C and at the restrictive temperature 29°C. Because genetic background has a profound impact on sleep [24], *Alk* mutants were backcrossed for five generations into a white (*w*) isogenic background, *iso31*, a line generated specifically for use in behavioral experiments [25]. At 18°C, control *iso31* and *Alk* flies had very similar sleep patterns. However, acute inhibition of *Alk* by switching the environmental temperature to 29°C drastically increased sleep in *Alk* flies as compared to *iso31*. In *iso31* flies, the shift to 29°C initially increased daytime sleep and decreased nighttime sleep, consistent with previous reports of increased siesta at higher temperatures [26]; overall sleep increased on the third day, but there was no net change in sleep over the three day period. Inhibition of *Alk* increased both day and night sleep. This temperature-sensitive sleep phenotype was reversed by lowering the temperature back to 18°C (Fig 1A). Quantification shows that *Alk* ^*ts/1*^ flies slept ~51.06±10.09% more than the control *iso31* flies during the high temperature shift (6 independent experiments, n = 87 for *iso31* and n = 80 for *Alk* ^*ts/1*^). Similarly, *Alk* ^*ts*^ homozygous flies and flies that harbor an *Alk* ^*ts*^ allele over a deficiency uncovering the *Alk* gene slept more than control flies at 29°C. There was no difference in total sleep amount between *Alk* ^*ts/1*^, *Alk* ^*ts*^, or *Alk* ^*ts/Def*^ flies, suggesting that the restrictive temperature completely abolished ALK^ts^ protein function (Fig 1B). Importantly, we were able to rescue the sleep phenotype of *Alk* mutants by re-expressing *Alk* transgenically (discussed below).

**Fig 2:**
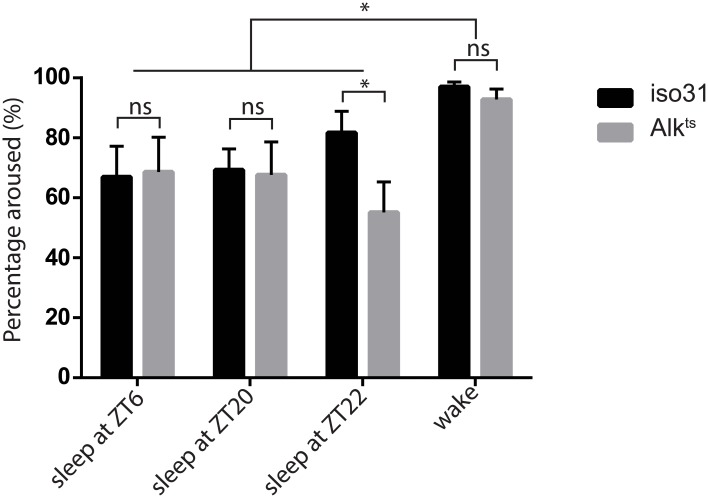
Inactivity of *Alk* ^*ts*^ mutants is due to a prolonged sleep state. To distinguish sleep from quiet wake, we subjected previously sleeping or awake flies to a mechanical stimulus at different times of day—ZT6, ZT20 and ZT22. Across all time points, two-way ANOVA found significant differences in the response to simulation between previous behavior states (p = 0.0071) but not between genotypes (p = 0.189). There was no interaction between behavior states and genotypes (p = 0.368). However, *Alk* flies were less arousable than wild type at ZT22, by Student’s t test. n = 4–5 trials of 26–32 flies of each genotype for all time points. The responses of previously awake flies were similar between the three time points and thus were pooled.

Though sleep profiles and sleep metrics produced by the standard single infrared beam sleep monitors are reliable and have been published widely, they sometimes overestimate sleep as they miss fly movement away from the infrared beam; the degree of error varies from one genotype to another [27,28]. We therefore assayed sleep in a new multi-beam sleep monitor (Trikinetics, MA), which provides an order-of-magnitude higher spatial resolution compared to the traditional single beam monitors. Measurements by multi-beam monitors validated our results from the single beam method, showing that *Alk* ^*ts/1*^ females had increased daytime and nighttime sleep at 29°C as compared to *iso31* control flies (Fig 1C and S2 Fig). However the long sleep phenotype was less pronounced when assayed with multi-beam monitors, with *Alk* ^*ts/1*^ female flies showing a 32.3±3.3% increase over *iso31* (3 independent experiments, 47 *iso31* flies and 48 *Alk* ^*ts/1*^flies). We also assayed sleep in *Alk* male flies with both monitor systems, and found that it was similarly increased (S3 Fig). Henceforth we focused our analysis on female flies, typically the gender studied in Drosophila sleep experiments. To exclude the possibility that the apparent increase in sleep was due to locomotion impairment, we measured waking activity, calculated as the average number of beam crossings per waking minute. We found that it did not account for the long sleep phenotype as it was reduced in both long-sleeping *Alk* ^*ts/1*^ flies as well as normal-sleep *Alk* ^*1/+*^ controls (Fig 1D). We also assayed the mobility of *Alk* flies in an independent negative geotaxis assay that measures the ability of flies to climb vertically when startled [29]. The response of *Alk* ^*ts/1*^ flies was indistinguishable from that of *iso31* flies (Fig 1E), suggesting that *Alk* ^*ts/1*^ flies have no gross motor defects. To confirm that the increased inactivity in *Alk* mutants is genuine sleep, as opposed to quiet wake, we assayed their arousability to a mechanical stimulus at different times of day (Fig 2). Similar percentages of previously sleeping *iso31* and *Alk* ^*ts*^ flies were aroused by the stimulus at ZT6 and ZT20, when most flies were sleeping. At ZT22, more iso31 flies were aroused than *Alk* ^*ts*^ flies, probably because *iso31* flies were transitioning from sleep to wake at this time point. Notably, a higher percentage of the previously awake flies exhibited activity after the stimulus than sleeping flies at all three time points, suggesting that arousal threshold was indeed higher in sleeping flies. We conclude that *Alk* mutants are bona fide long sleepers, suggesting that ALK functions to inhibit sleep or promote wake.

### *Alk* mutants show a normal sleep homeostatic response

**Fig 3:**
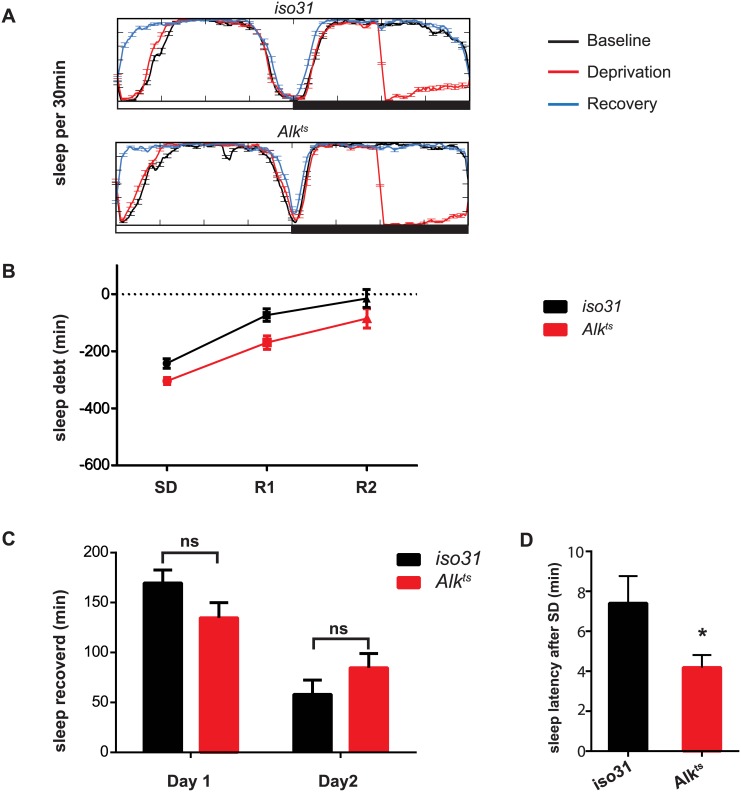
Homeostatic response to sleep loss in *Alk* mutants. A) Sleep profiles for the baseline, deprivation and recovery day were plotted against each other to show sleep deprivation and sleep rebound. The experiment was conducted entirely at 29°C. *Iso31* and *Alk* ^*ts*^ similarly show an increase in sleep in the morning following sleep deprivation. B) Time course of recovery of sleep lost following deprivation. C) Minutes of sleep recovered on the first and second recovery day were compared between *iso31* and *Alk* ^ts^ flies. 2-tailed Student’s t-test shows no significant difference between *iso31* and *Alk* ^*ts*^ flies. D) Sleep latency after deprivation on recovery day one. *p<0.05. N = 31 for iso31. N = 29 for *Alk* ^*ts*^.

Following a period of sleep deprivation, flies, like other animals, show a homeostatic response in the form of increased sleep [30]. We wondered whether this homeostatic regulation was disrupted in *Alk* mutants. We found that after 6 hours of sleep deprivation by mechanical stimulation, both *iso31* and *Alk* ^*ts*^ flies increased sleep the following morning (Fig 3A). However, sleep-deprived *Alk* ^*ts*^ flies fell asleep faster than *iso31* controls, suggesting sleep pressure was higher in *Alk* ^*ts*^ flies (Fig 3A and 3D).

### Inhibition of *Alk* in various brain regions has differential effects on sleep

**Fig 4:**
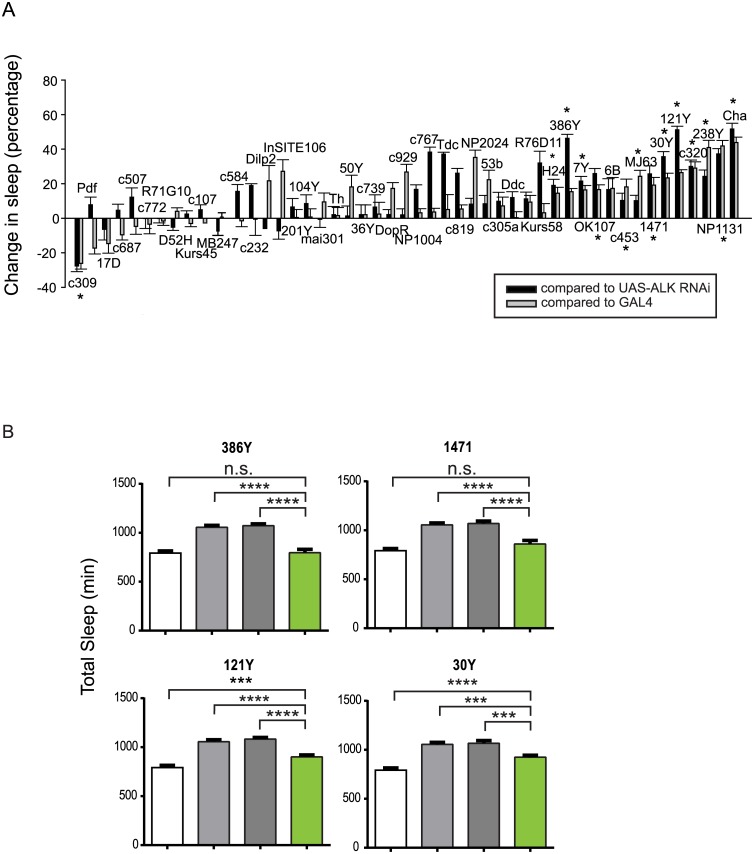
*Alk* is required in a subset of CNS neurons to regulate sleep. A) A targeted *Alk* RNAi screen with CNS Gal4 drivers to identify regions where ALK acts to regulate sleep. The graph shows the percentage changes in total sleep as a result of expressing *Alk* RNAi compared to controls *UAS-Alk RNAi/+; Dcr2/+* (black bars) and *Gal4/+* (grey bars). The difference in sleep amount between the experimental group and each control group was divided by the amount of control sleep to calculate net percentage change. Bars represent pooled data from 2–4 experiments for each genotype and show means ±SEM (n = 8–58 for experimental groups and each Gal4 control group. N = 131 for the *UAS-Alk RNAi* control). Daily sleep was averaged over 3 days. One-way ANOVA and post hoc analysis were done to compare total sleep in the *Alk* RNAi-expressing group to *UAS-Alk RNAi* and *Gal4* control groups. * indicates that total sleep of the RNAi expressing group is significantly different from those of both control group. B) The long sleep phenotype of *Alk* ^*ts*^ at the restrictive temperature can be rescued by expressing *Alk* in sub-regions of the brain. Genotypes for the four bars: *iso31* (white), *Alk* ^*ts*^, *UAS-Alk*/*Alk* ^*ts*^; *Tub-Gal80* ^*ts*^/+ (light grey), *Alk* ^*ts*^ *; Gal4*/+ (dark grey), *Alk* ^*ts*^, *UAS-Alk*/*Alk* ^*ts*^; *Gal4*/*tub-Gal80* (green). The white and light grey controls are the same in these graphs. N = 21–46. Total sleep amounts were averaged for 3 days at 29°C. One-way ANOVA and post hoc Turkey’s test were performed for all pairwise comparisons. In all groups, grey bars (*Alk* ^*ts*^ controls) are significantly higher than the white bar (****p<0.0001), which is not indicated in the graph.

Similar to the expression pattern of the mouse ALK gene, the *Drosophila Alk* gene is extensively expressed in the developing and adult nervous system [12,14,23]. Its adult expression includes the mushroom body, the protocerebral bridge, the antennal lobes, the suboesophageal ganglion, the medial bundle and lateral horns [14]. To locate the sleep regulatory function of *Alk* in the brain, we carried out a brain mini-screen using a series of GAL4 lines to drive UAS-*Alk* RNA interference (RNAi) in brain circuits. Inducing *Alk* RNAi with a pan-neuronal driver, *elav*-GAL4, reduced *Alk* mRNA level to ~35% of that of control flies and produced longer sleep, suggesting that ALK functions in neurons (S4 Fig). We then screened over 40 GAL4 drivers with diverse neuronal expression patterns (S1 Table and S5 Fig), including those with expression patterns in the known sleep/wake regulating regions [31–39]. 13 GAL4 lines significantly increased sleep relative to controls, when driving *Alk* RNAi, while c309-Gal4-driven expression of *Alk* RNAi decreased sleep (Fig 4A). Of the identified sleep-promoting GAL4 lines, most are broadly expressed, overlapping in several regions of the brain, such as the mushroom body, the ellipsoid body, and the pars intercerebralis. However, inhibiting *Alk* specifically in the pars intercerebralis (InSITE106, Kurs58 and Dilp2-GAL4), or the ellipsoid body (c819, c232 and c107) did not alter total sleep. The amounts of sleep increase as well as the sleep profiles were different between different GAL4 lines (S6 Fig). Most lines showed increased daytime sleep as well as nighttime sleep. However, lines 1471, c320 and 386Y mainly increased daytime sleep and 7Y mostly increased night-time. 386Y caused a delay in sleep at the beginning of the night while driving an increase at other times. c320 increased sleep immediately after lights-on in the morning. These sleep patterns were consistent across repeated experiments.

We found that the long sleep phenotype of *Alk* mutants could be rescued by restoring functional ALK with targeted drivers that we identified through the RNAi screen. To circumvent the developmental effects of overexpressing *Alk*, we expressed *Alk* in adult *Alk* ^*ts*^ mutants only during a period of restrictive temperature at 29° by combining GAL4s with a temperature-sensitive form of GAL80 (tub-Gal80^ts^) [40]. We tested 386Y, 7Y, 121Y, 1471, 30Y and MJ63, all of which induce longer sleep when driving *Alk* RNAi. We found that 386Y and 1471 fully rescued the long sleep phenotype of *Alk* ^*ts*^ at high temperature, while 30y and121y produced a partial rescue (Fig 4B). 7Y and MJ63, however, did not rescue the long sleep of *Alk* ^*ts*^, and neither did any of the drivers that yielded no phenotype with *Alk* RNAi (S7 Fig). We infer that while *Alk* expression is necessary, it may not be sufficient for sleep regulation in regions of 7Y and MJ63 expression. The rescue results further confirm that the long sleep phenotype is specific to the *Alk* gene and involves specific brain regions.

### *Alk* negatively regulates sleep in a subset of mushroom body neurons

**Fig 5:**
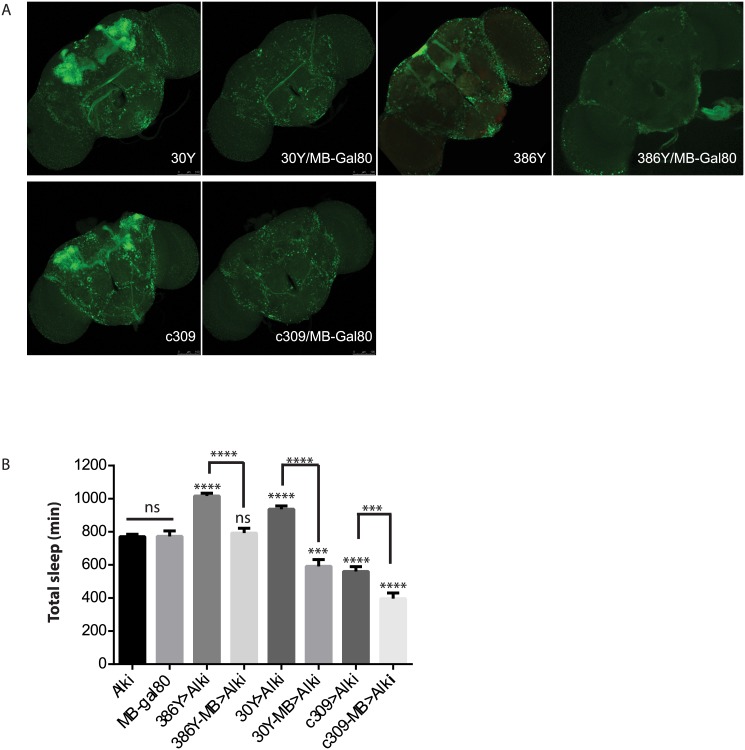
ALK functions in the mushroom body to inhibit sleep. A) MB-Gal80 eliminates mushroom body expression from 30Y, 386Y and c309 Gal4s. B) *Alk* RNAi-induced sleep increase is suppressed by MB-Gal80. GAL4-MB represents combination of GAL4 and MB-Gal80. * above bars indicate significant differences from both UAS-Alk RNAi and MB-Gal80 controls. Brackets show comparisons between Gal4>Alk RNAi and Gal4-MB>Alk RNAi. ns, not significant, ***p<0.001; ****p<0.0001, by One-way ANOVA and Turkey’s *post hoc* analysis. n = 18–49. In combination with 30y, MB-Gal80 decreases sleep below control levels. With C309, MB-Gal80 further decreases sleep. UAS-RNAi and MB-gal80, and 386y-MB are not statistically different.

Prompted by the extensive mushroom body expression of most driver hits in our screen, we focused on the function of ALK in the mushroom bodies (MB). When *Alk* RNAi was excluded from the MB by combining GAL4s with a mushroom body-specific Gal80 transgene (MB-Gal80)[41](Fig 5), it eliminated the sleep increase induced by 386Y and 30Y, suggesting that *Alk* function is required in mushroom body neurons labeled by these two drivers. In fact, when 30Y was combined with MB-Gal80, it decreased sleep to below those of controls. c309, a driver that caused short sleep with *Alk* RNAi, also has extensive mushroom body expression. Driving *Alk* RNAi with c309/MB-Gal80, however, decreased sleep further compared to *Alk* RNAi driven by c309. These results indicate that the sleep-increasing effects of *Alk* deficiency occur mainly in the MBs and with 30Y and c309 they are countered by sleep-inhibiting influences of *Alk* knockdown outside the mushroom body. We thus tested several drivers that have localized expression in the mushroom body and little expression elsewhere, but did not observe any increase in sleep (Fig 4). Of these three drivers, 17D innervates the core of α/β lobes; D52H has sexually dimorphic expression with strong expression in the α/β and the main γ lobe in males but faint dorsal γ expression in the females, which is the gender we used for sleep assays; R71G10 preferentially innervates the γ lobe and R76D11 has strong expression in both α/β and γ; c305a, which innervates α′/β′ lobes in addition to cells in other brain regions [42,43]. Given that of the positive drivers, H24, NP1131 and 1471 label Kenyon cells that project exclusively in the γ lobes, we hypothesize ALK functions to inhibit sleep in a subset of γ lobe neurons that are not targeted by R71G10 and R76D11.

### Sleep defects in *Nf1* mutants

**Fig 6:**
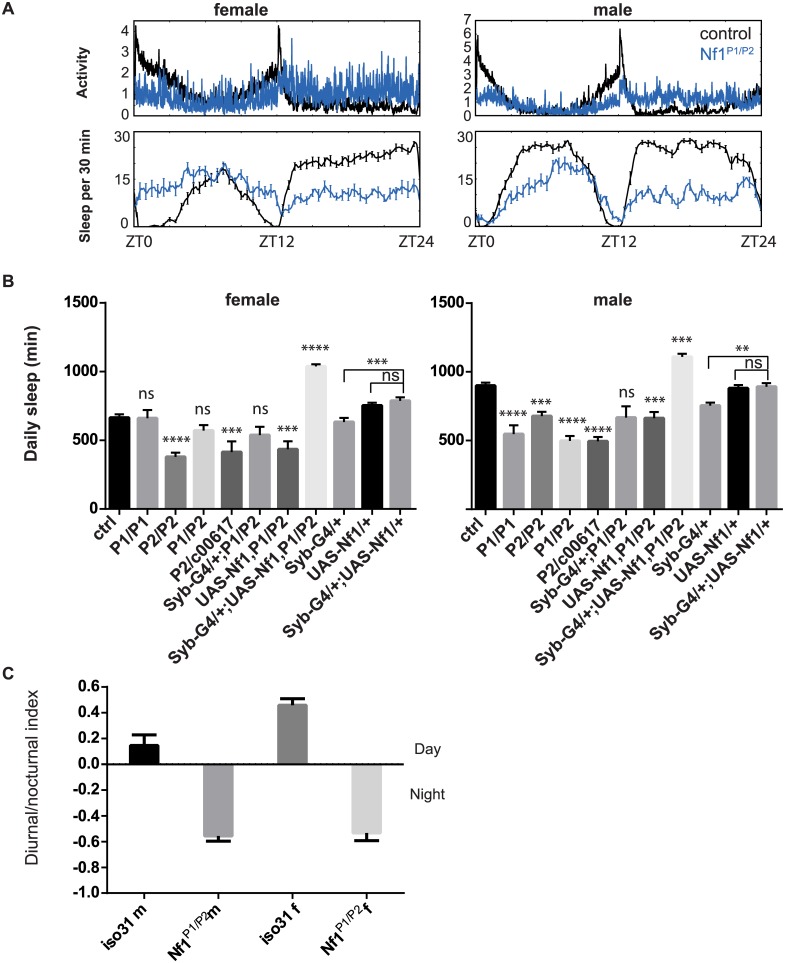
Sleep reduction in *Nf1* mutants. A) Averaged sleep profiles of control *iso31* (black) and *Nf1* ^*P1/P2*^ (blue) mutant flies at 25°C. B) Average daily sleep for control *iso31*, *Nf1* mutants and *Nf1* flies with transgenic *Nf1* gene rescue at 25°C. Comparisons were done with one-way ANOVA and post-hoc Turkey’s test. * indicates significant difference from iso31 control. Error bars are SEM. N = 5–16. Ns, not significant. ***p<0.001, ****p<0.0001. *Nf1* transgenic expression in wild type background was done in a separate experiment and their sleep quantities were compared with the respective controls. C) *Nf1* mutants exhibit nocturnal hyperactivity. Diurnal/nocturnal index was calculated as (total activity during the day) − (total activity during the night)/(total activity), averaged over a 3-d period per fly.

Genetic interactions between *Alk* and *Nf1* in growth and learning processes led us to investigate whether *Nf1* is also required for sleep regulation. Interestingly, a prevalence of sleep disturbances has recently been reported in NF1 patients [44,]. We detected considerable variability in total sleep amount in *Nf1* mutant flies across experiments. We tested three *Nf1* alleles, *Nf1* ^*P1*^, *Nf1* ^*P2*^, and *Nf1* ^*c00617*^. *Nf1* ^*P1*^ and *Nf1* ^*P2*^ alleles are both assumed to be null because neither expresses NF1 protein and homozygous flies have similar defects in locomotor activity rhythms, body size and learning [22,46, 47]. Although the average total sleep of male flies harboring any two *Nf1* alleles was significantly less than that of control flies, we did not observe a consistent difference between *Nf1* mutant and control female flies (Fig 6). While *Nf1* ^*P2/c00617*^ female flies slept less than controls, sleep amounts in *Nf1* ^*P1/P2*^ female flies were generally not different from those of control flies. Furthermore, *Nf1* ^*P1*^ and *Nf1* ^*P2*^ female flies did not consistently show sleep reduction as compared to *iso31* controls in separate experiments. Similar discrepancies were found when sleep was assayed with the multi-beam sleep monitors. However, both *Nf1* male and female flies exhibited nocturnal hyperactivity (Fig 6A and 6C), which resulted in an increase in daytime sleep and a decrease in nighttime sleep. *Nf1* mutants also showed consistent defects in sleep consolidation. Both daytime sleep and nighttime sleep were highly fragmented in *Nf1* males, such that the average sleep bout duration was reduced and the number of sleep bouts increased (S8 Fig). While reductions in average bout duration did not reach significance in females, increased numbers of bouts suggest deficits in sleep maintenance and consolidation. Rescuing *Nf1* with pan-neuronal expression of a *UAS-Nf1* transgene increased sleep of *Nf1* mutants and in fact caused a long sleep phenotype compared to wild-type controls. This did not result from ectopic expression of the transgene as expressing the same UAS-*Nf1* transgene in wild-type flies had no effect (Fig 6B). Interestingly, sleep increase was seen with *Nf1* rescue in both male and female flies, suggesting that effects of *Nf1* on sleep are quite complex.

### *Alk* interacts functionally with *Nf1* to regulate sleep

**Fig 7:**
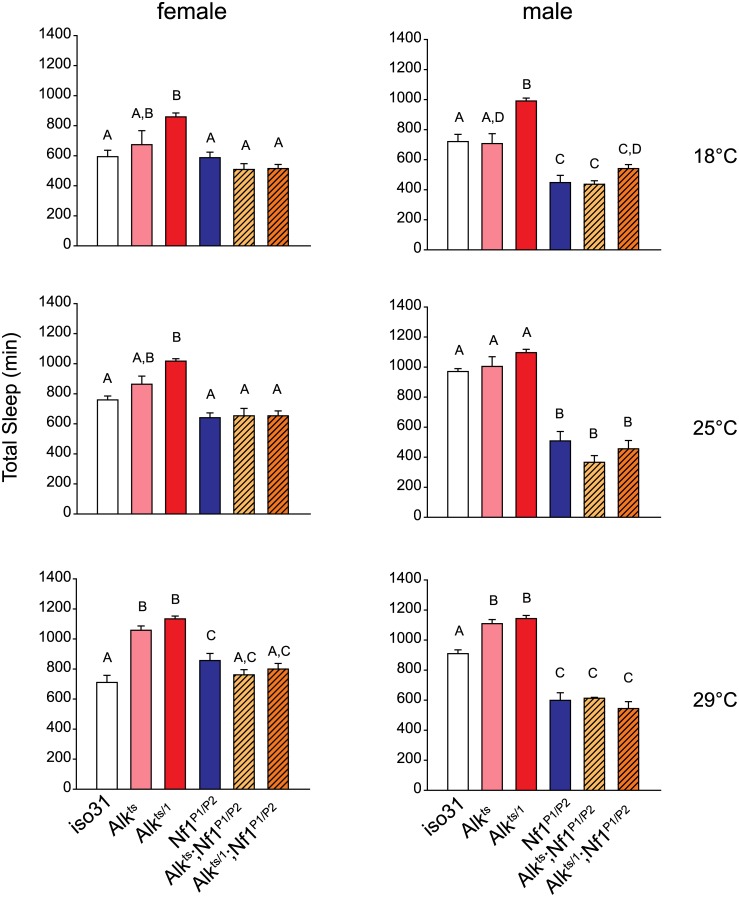
*Nf1* mutations suppress the long-sleep phenotype of *Alk* mutants. Bar graphs show total sleep for *iso31* controls, *Alk* mutants, *Nf1* ^*P1/P2*^ mutants, and *Alk; Nf1* double mutants. One-way ANOVA and Mann-Whitney *post hoc* analysis was performed to compare groups in each condition. In each graph, groups with the same alphabet label on top are not statistically different from each other; groups with different labels are statistically different (p<0.05). For experiments at 18°C and 25°C, n = 5–16. For 29°C experiment, n = 10–37. All flies were raised at 18°C. Activities were monitored at 18°C, 25°C and 29°C in independent experiments.

We then investigated whether *Alk* and *Nf1* interact to regulate sleep. We chose to test *Alk* with the *Nf1* ^*P1/P2*^ allelic combination because *Nf1* ^*P1/P2*^ females have normal amount of sleep and so any sleep suppression in the double mutants would not be confounded by additive effects of short-sleeping *Nf1* mutants. To sensitize the assay, we compared sleep in *Alk* ^ts^, *Alk* ^*ts/1*^ or *Nf1* ^*P1/P2*^ single mutants and *Alk* ^*ts*^ *;Nf1* ^*P1/P2*^ and *Alk* ^*ts/1*^ *;Nf1* ^*P1/P2*^ double mutants at three different temperatures that render different dosages of functional ALK. Interestingly, total amounts of sleep in *Alk*;*Nf1* double mutant flies were less than those of flies deficient for *Alk* alone, and not different from *Nf1* single mutants or *iso31* control flies (Fig 7). We found that regardless of the severity of the *Alk* alleles, *Nf1* ^*P1/P2*^ completely suppressed the long sleep phenotype. As another piece of evidence for genetic interaction, we found that *Nf1* ^P1/P2^ also suppressed the long sleep phenotype caused by *Alk* pan-neural RNAi (S9 Fig). These results suggest that *Nf1* interacts with *Alk* in a sleep regulating circuit.

### *Alk* does not interact with *Nf1* to control circadian rhythms

**Fig 8:**
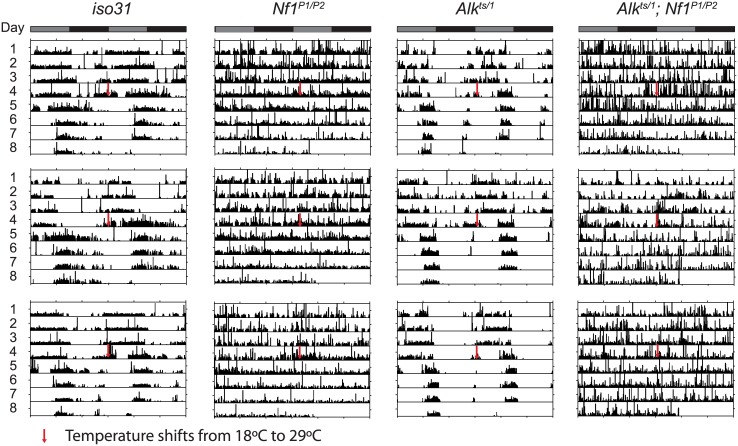
*Alk* does not interact with *Nf1* to control circadian rhythms. Representative activity graphs for each genotype are shown with activity double-plotted (2 day/night cycles). Gray and black bars at the top indicate subjective days and nights. Flies were raised and entrained at 18°C, monitored for 4 days in constant darkness at 18°C and then at 29°C for 4 days. Upon the temperature shift, *iso31* and *Alk* ^*ts/1*^ flies manifest a phase shift in their activity rhythms. *Nf1* ^*P1/P2*^ and *Alk* ^*ts/1*^ *;Nf1* ^*P1/P2*^ double mutants are arrhythmic.

*Nf1* is part of the circadian output pathway that controls rest: activity rhythms [22]. As *Alk* was found to interact with *Nf1* in sleep regulation, as well as in growth and learning, we asked whether *Alk* is required also for circadian rhythms and whether *Alk* and *Nf1* interact in circadian pathways. We found that *Alk* ^*ts/1*^ trans-heterozygotes raised at 18°C maintained locomotor activity rhythms at the restrictive temperature of 29°C in constant darkness (Fig 8 and Table 1), indicating *Alk* is not required to maintain circadian activity. Indeed, the FFT values, a measure of rhythm strength, of *Alk* ^*ts/1*^ and *Alk* ^*ts/Def*^ flies were higher at 29°C than at 18°C, suggesting that loss of *Alk* may actually improve rhythms rather than disrupt them. However, inhibiting *Alk* failed to rescue the circadian defects in *Nf1* flies: *Alk* ^*ts/1*^; *Nf1* ^*P1/P2*^ double mutant were arrhythmic, just like *Nf1* single mutants. To exclude a requirement for *Alk* in the development of circadian circuits, we also tested *Alk* ^*ts*^ homozygous flies raised at 25°C, at which temperature *Alk* ^*ts*^ flies have moderate lethality [19]. The partial reduction in ALK function throughout development did not cause arrhythmia nor did it suppress arrhythmia in *Nf1* ^*P1/P2*^ flies (Table 1). These results suggest that *Alk* does not function in the circadian output circuit regulated by *Nf1*.

**Table 1:** Circadian rhythmicity of Alk and Nf1 mutants.

Genotype	18°C*			29°C*			25°C†		
	Period (hr)	FFT	% R (n)	Period (hr)	FFT	% R (n)	Period (hr)	FFT	% R (n)
*iso31*	23.26±0.19	0.073±0.005	100.0 (16)	23.80±0.04	0.108±0.011	100.0 (16)	23.41±0.06	0.051±0.005	100.0(16)
*Alk* ^*ts*^	23.70±0.25	0.064±0.008	93.3 (15)	23.79±0.04	0.087±0.011	100.0 (15)	23.60±0.06	0.052±0.007	100.0(15)
*Alk* ^*ts/1*^	23.45±0.14	0.063±0.010	86.7 (15)	23.90±0.01	0.124±0.010	100.0 (15)			‡
*Alk* ^*ts/Def*^	23.26±0.24	0.077±0.011	100.0 (9)	23.77±0.04	0.124±0.011	100.0 (9)			‡
*Nf1* ^*P1/P2*^			0 (16)			0 (16)			0 (24)
*Alk* ^*ts/1*^ *;Nf1* ^*P1/P2*^			0 (11)			0 (11)			0 (23)

*Flies were raised and entrained at 18. Their activities were monitored in DD at 18°C for 4 days and at 29°C for another 4 days.

^†^Flies were raised and tested at 25°C.

^‡^ *Alk* ^*ts/1*^ and *Alk* ^*ts/Def*^ flies are not viable when raised at 25°C.

## Discussion

Though a few studies implicate *Alk* orthologs in regulating behaviors such as decision-making, cognition, associative learning and addiction, most functional studies demonstrate various developmental roles for *Alk* [14,20,21,48–50]. We acutely induce a long-sleep phenotype by taking advantage of a temperature-sensitive allele, *Alk* ^*ts*^, revealing that *Alk* regulates sleep directly rather than through developmental processes. We also show mutations in *Nf1*, a gene encoding a GAP that regulates the Ras/ERK pathway activated by ALK, causes a sexually dimorphic short-sleep phenotype. Thus we establish a novel *in vivo* function for both *Alk* and *Nf1* and show they interact with each other to regulate sleep.

### Role of *Alk* in sleep

Many downstream signaling pathways have been proposed for ALK, among them Ras/ERK, JAK/STAT, PI3K and PLCγ signaling [11]. ERK activation through another tyrosine receptor kinase Epidermal growth factor receptor (EGFR) has been linked to increased sleep [36,51], while here we show that *Alk*, a positive regulator of ERK, inhibits sleep. We note that ERK is a common signaling pathway targeted by many factors, and may have circuit- specific effects, with different effects on sleep in different brain regions. Indeed, neural populations that mediate effects of ERK on sleep have not been identified. The dose of ALK required for ERK activation might also differ in different circuits. Region-specific effects of *Alk* are supported by our GAL4 screen, in which down-regulation of *Alk* in some brain regions even decreased sleep. The overall effect, however, is to increase sleep, evident from the pan-neuronal knockdown. We found that the mushroom body, a site previously implicated in sleep regulation and learning, requires *Alk* to inhibit sleep. Interestingly, the expression patterns of *Alk* and *Nf1* overlap extensively in the mushroom body [14], suggesting that they may interact here to regulate both sleep and learning. However, it was previously shown that *Alk* activation in the mushroom body has no effect on learning [14]. The mushroom body expression in that study was defined with MB247 and c772, both of which also had no effects on sleep when driving *Alk* RNAi (Fig 4). The spatial requirement for *Nf1* in the context of learning has been disputed in previous studies with results both for and against a function in the mushroom body [14,52]. The discrepancies between these studies could result from: 1) varied expression of different drivers within lobes of the mushroom body, with some not even specific to the mushroom body; 2) variability in the effectiveness and specificity of MB-Gal80 in combination with different GAL4s. We confirmed that our MB-Gal80 manipulation eliminated all mushroom body expression and preserved most if not all other cells with 30Y, 386Y and c309. Future work will further define the cell populations in which *Alk* and *Nf1* interact to affect sleep.

### Role of *Nf1* in sleep regulation

We observed a substantial sleep decrease in *Nf1* male flies compared to control flies. However, sleep phenotypes in *Nf1* female flies are inconsistent. It is unlikely that unknown mutations on the X chromosome cause the short-sleeping phenotype because our 7 generation outcrosses into the control *iso31* background started with swapping X chromosomes in *Nf1* ^*P1*^ and *Nf1* ^*P2*^ male flies with those of *iso31* flies. In support of a function in sleep regulation, restoring *Nf1* expression in neurons of *Nf1* mutants reverses the short sleep phenotype to long sleep in both males and females. This does not result from ectopic expression of the transgene as expressing the same UAS-*Nf1* transgene in wild-type flies has no effect. We hypothesize that *Nf1* promotes sleep in some brain regions and inhibits it in others, and sub-threshold levels of *Nf1*, driven by the transgene in the mutant background, tilt the balance towards more sleep. As reported here, *Alk* also has differential effects on sleep in different brain regions, as does protein kinase A [33], thus such effects are not unprecedented. We also note severe sleep fragmentation in *Nf1* mutants, which suggests that they have trouble maintaining sleep.

The sex-specific phenotypes of *Nf1* mutants may reflect sexually dimorphic regulation of sleep. A recently published genome-wide association study of sleep in *Drosophila* reported that an overwhelming majority of single nucleotide polymorphisms (SNPs) exhibit some degree of sexual dimorphism: the effects of ~80% SNPs on sleep are not equal in the two sexes [53]. Interestingly, sex was found to be a major determinant of neuronal dysfunction in human NF1 patients and *Nf1* knock-out mice, resulting in differential vision loss and learning deficits [54]. The sex-dimorphic sleep phenotype in *Nf1* flies provides another model to study sex-dimorphic circuits involving *Nf1*. Interestingly, a prevalence of sleep disturbances have recently been reported in NF1 patients [44,45], suggesting that NF1 possibly play a conserved function in sleep regulation.

### Links between plasticity and sleep

An attractive hypothesis for a function of sleep is that plastic processes during wake lead to a net increase in synaptic strength and sleep is necessary for synaptic renormalization [3]. There is structural evidence in *Drosophila* to support this synaptic homeostasis hypothesis (SHY): synapse size and number increase during wake and after sleep deprivation, and decrease after sleep [55]. However, little is known about the molecular mechanisms by which waking experience induces changes in plasticity and sleep. FMRP, the protein encoded by the *Drosophila* homolog of human fragile X mental retardation gene FMR1, mediates some of the effects of sleep/wake on synapses [55,56]. Loss of *Fmr1* is associated with synaptic overgrowth and strengthened neurotransmission and long sleep. Overexpressing *Fmr1* results in dendritic and axonal underbranching and short sleep. More importantly, overexpression of Fmr1 in specific circuits eliminates the wake-induced increases in synapse number and branching in these circuits. Thus, up-regulation of FMR accomplishes a function normally associated with sleep.

We hypothesize that *Alk* and *Nf1* similarly play roles in synaptic homeostasis. They are attractive candidates for bridging sleep and plastic processes, because: 1) *Alk* is expressed extensively in the developing and adult CNS synapses [14,57]. In particular, both *Alk* and *Nf1* are strongly expressed in the mushroom body, a major site of plasticity in the fly brain. 2) Functionally, postsynaptic hyperactivation of *Alk* negatively regulates NMJ size and elaboration [19]. In contrast, *Nf1* is required presynaptically at the NMJ to suppress synapse branching [58]. 3) *Alk* and *Nf1* affect learning in adults and they functionally interact with each other in this process [14]. It is tempting to speculate that in *Alk* mutants, sleep is increased to prune the excess synaptic growth predicted to occur in these mutants. Such a role for sleep is consistent with the SHY hypothesis. The SHY model would predict that *Alk* flies have higher sleep need, which is expected to enhance rebound after sleep deprivation. While our data show equivalent quantity of rebound in *Alk* mutants, we found that they fall asleep faster than control flies the morning after sleep deprivation (Fig 3), suggesting that they have higher sleep drive. Increased sleep need following deprivation could also be reflected in greater cognitive decline, but this has not yet been tested for *Alk* mutants. We note that *Nf1* mutants have reduced sleep although their NMJ phenotypes also consist of overbranched synapses [58,59]. We postulate that their sleep need is not met and thus results in learning deficits. Clearly, more work is needed to test these hypotheses concerning the roles of *Alk* and *Nf1* in sleep, learning, and memory circuits.

## Materials and Methods

### Fly lines

The following lines were used previously in the lab [33, 37, 60]: Elav-Gal4, 201Y-GAL4, c739-GAL4, 238Y-GAL4, c309-GAL4, Kurs58-GAL4, Pdf-Gal4, H24-GAL4, c507-GAL4, 30Y-GAL4, 50Y-GAL4, MJ63-GAL4, c232-GAL4, 104Y-GAL4, 17D-GAL4, Dilp2-Gal4, Tdc2-Gal4, TH-Gal4, Mai301-GAL4, c767-GAL4, 1471-GAL4, ok107-GAL4, c929-GAL4, 53b-GAL4, c320-GAL4 and UAS-GFP.NLS. elav-GAL4; Dcr2 (25750), c305a-GAL4 (30829), c107-GAL4(30823), c819-GAL4 (30849), 121Y-GAL4 (30815), 7Y-GAL4 (30812), 36Y-GAL4 (30819), 386Y-GAL4 (25410), c584-GAL4 (30842), DopR-GAL4 (19491), Ddc-GAL4 (7009), R71G10-GAL4 (39604) and R76D11-GAL4 (39927) were ordered from the Bloomington Drosophila Stock Center. NP1131-GAL4 (103898), NP1004-GAL4 (112440) and NP2024-GAL4 (112749) were ordered from the Drosophila Genetic Resource Center. Tub-GAL80^ts^ [40], MB-Gal80 [41], D52H-GAL4 [42], c687-GAL4 [61], 6B-GAL4 [62], InSITE106-GAL4 [63],Cha-GAL4 [64], Kurs45-GAL4 [65] were gifts. *Nf1* ^*P1*^ and *Nf1* ^*P2*^ alleles were reported [22] and were outcrossed into an *iso31* background for 7 generations. *Alk* ^*ts*^/CyO was a gift from Dr. J Weiss [13] and was outcrossed into an *iso31* background. *Alk* ^*1*^ */CyO* and *UAS-Alk/CyO* were gifts from Dr. R Palmer and both were outcrossed into an *iso31* background [13,16]. The deficiency line uncovering the *Alk* gene, *Alk* ^*Def*^ (7888)/CyO, was ordered from Bloomington. *Alk* RNAi (11446) and *UAS-Dcr2*(60008) were ordered from the Vienna Drosophila Resource Center.

### Immunohistochemistry

Reporter GFP expression driven by GAL4 lines was visualized through whole-mount brain immunofluorescence as previously described [60]. Rabbit anti-GFP (Molecular Probes A-11122) 1:1000 and Alex Fluor 488 Goat anti-rabbit (Molecular Probes A-11008) 1:500 were used.

### Sleep assays

Sleep was monitored as described previously [37]. Flies were raised and kept on a 12h:12h light/dark (LD) cycle at 18°C or 25°C as stated. 3–7 day old flies were loaded into glass tubes containing 5% sucrose and 2% agar. Locomotor activity was monitored with the Drosophila Activity Monitoring System (Trikinetics, Waltham MA), or when indicated with multi-beam monitors (Trikinetics, Waltham MA) that generate 17 infrared beams. Data were analyzed with Pysolo software [5]. For sleep assays with temperature shifts, total sleep amount was averaged for 3 d at the lower temperature before the shift and 3 d at the high temperature. For sleep deprivation experiments, flies were monitored for a baseline day and then sleep deprived on the second day for 6 hours from *Zeitgeber Time* (ZT) 18 to ZT24 during the night. Sleep was continually monitored for 2 recovery days. Mechanical sleep deprivation was accomplished using a Trikinetics vortexer mounting plate, with shaking of monitors for 2 seconds randomly within every 20 second window for 6 hours. The arousal threshold assay was described previously [66]. A 12oz rubber weight was dropped from 2-inch height onto a rack supporting large DAMS monitors at ZT20. Flies with no activity 5 min before a stimulus and exhibited beam crossings within 5 min after the light pulse were recorded as “aroused”.

### Rest-Activity rhythm analysis

Individual male flies were loaded into glass tubes containing 5% sucrose and 2% agar. Locomotor activity was monitored with the Drosophila Activity Monitoring System (Trikinetics, Waltham MA), and analyzed with Clocklab software (Actimetrics, Wilmette). To evaluate ALK’s role in maintaining adult rhythms, all genotypes were raised at 18°C to avoid inactivating ALK during development. 3–7 d old flies were then loaded into glass tubes and entrained for 3 d to a 12h:12h LD cycle, followed by 4 days in constant darkness at 18°C and then 4 days in constant darkness at 29°C. Rhythmicity analysis was performed for each 4 d period. In a separate experiment, *iso31*, *Nf1* ^*P1/P2*^, *Alk* ^*ts*^ and *Alk* ^*ts*^ *;Nf1* ^*P1/P2*^ flies were raised and tested at 25°C to evaluate whether inhibiting *Alk* during development affect rest-activity rhythm. A fly was considered rhythmic if it met 2 criteria: 1) displayed a rhythm with 95% confidence using χ2 periodogram analysis, and 2) a corresponding FFT value above 0.01 for the determined period length.

### Negative geotaxis

The negative geotaxis assay was adapted from (Barone MC and Bohmann D 2013). Please see supplemental methods for details.

### Statistics

Data were analyzed and plotted with SigmaPlot and GraphPrism software. One-way ANOVA analysis was done to reveal differences between genotypes in the same experiments and pairwise comparison between genotypes were done with post-hoc analysis as indicated in the figures. ns, not significant; **, p<0.01; ***, p<0.001; ****, p<0.0001.

## Supporting Information

S1 Fig Pupae sizes of *Alk* ^*ts/1*^ mutants are normal at 18°C. P = 0.074. n = 15 for *iso31* and 9 for *Alk* ^*ts/1*^. Measurement was done with an EZ mic electronic micrometer.
(EPS)

S2 Fig *Alk* ^*ts/1*^ mutant females have increased sleep during both day and night. A) Sleep profiles in 30-min intervals for *iso31* (black), *Alk* ^*1/+*^ (blue) and *Alk* ^*ts/1*^ (green) flies as measured at 29°C with multi-beam monitors. The white and the black bars below the x-axis indicate 12-hour light and 12-hour dark periods. B) Amounts of daytime and nighttime sleep for each genotype. In this figure and subsequent figures, error bars represent SEM. Significant differences were found between genotypes during the day (one-way ANOVA, p<0.0001) and during the night (p = 0.0002). Post-hoc pairwise comparison was done with Turkey’s test. N = 15–16. **p<0.01, ***p<0.001, ****p<0.0001.
(EPS)

S3 Fig *Alk* mutant males have increased sleep. A) The restrictive temperature of 29°C reversibly increases sleep in *Alk* ^*ts/1*^ males. The averaged sleep profiles, plotted as average amounts of sleep in every 30-minute period, are shown for *iso31* and *Alk* ^*ts/1*^ flies. The white and the grey columns mark periods of day and night, respectively. N = 16. B) Quantification of average daily sleep of control and *Alk* mutants measured by single beam monitors. Total sleep at 18°C was calculated as the average of 3 days before the temperature shift to 29°C and was found to be not different between genotypes. Total sleep amounts at 29°C were calculated as the average of the 3 days following the temperature shift and are significantly different between genotypes (One-way ANOVA, p<0.0001). Asterisk* signifies difference from *iso31* control. *, p<0.05; ***,p<0.001. Error bars are SEM. N = 13–16. C) Quantification of average daily sleep of control and *Alk* mutants measured by multi-beam monitors. 2-tailed t-tests were performed to compare *Alk* ^*ts/1*^ and control flies. At 18°C, p = 0.007; at 29°C, p<0.0001. *iso31*, n = 29; *Alk* ^ts/1^, n = 27.
(EPS)

S4 Fig Pan-neuronal expression of *Alk* RNAi increases sleep. A) Quantitative real time PCR analysis confirms knock-down of *Alk* mRNA levels relative to controls with pan-neuronal *elav-gal4* in fly heads. B) Averaged sleep profile for flies that express *Alk* RNAi pan-neuronally (green), and two control groups: *UAS-AlkRNAi* (black) and *elav-GAL4* (blue). N = 16 for each genotype.
(EPS)

S5 Fig Expression patterns of GAL4 lines that induce a sleep phenotype with *Alk* RNAi. Gal4 expression in the brain was visualized with an nls-GFP reporter and stained with GFP antibody.
(EPS)

S6 Fig Effects of region-specific *Alk* knockdown on sleep profiles. Averaged sleep profiles of *Alk* RNAi-expressing flies plotted against their respective controls. GAL4 control flies were offspring of *GAL4* and *iso31*. UAS-Alk RNAi control flies were offspring of *UAS-Alk RNAi* and *iso31*.
(EPS)

S7 Fig Restoring ALK expression with some GAL4s fails to rescue the long-sleep phenotype of *Alk* mutants. Loss of ALK activity in the *Alk* ^*ts*^ mutants and expression of a transgenic UAS-Alk by different GAL4s (in combination with tub-GAL80^ts^) were simultaneously induced at 29°C. Total sleep amounts were averaged for 3 days at 29°C. In controls that harbor only UAS-Alk (black bar) or individual GAL4 drivers (grey bars) total sleep is significantly longer than in *iso31* (white bar). There are no significant differences between *Alk* ^*ts*^ flies expressing UAS-*Alk* with different GAL4 drivers (green bars) and their controls. One-way ANOVA and Mann-Whitney *post hoc* analysis were performed. n = 11–53.
(EPS)

S8 Fig Sleep is fragmented in *Nf1* mutants. Sleep was assayed with multi-beam monitors. The duration and number of sleep episodes during the day and night were analyzed with Pysolo software. 2-tailed Student t-tests were performed between *iso31* and *Nf1* ^*P1/P2*^ flies. Error bars are SEM. n = 15–16.
(EPS)

S9 Fig *Nf1* mutations suppress the long sleep phenotype caused by *Alk* RNAi. Experiment was done at 25°C and used female flies. Pan neuronal *Alk* RNAi was driven with *P{nSyb-GAL4}*. n = 4–8. **, p<0.01. ***, p<0.001.
(EPS)

S1 Text Supplementary methods. (DOCX)

S1 Table GAL4 drivers used in the screen. (DOCX)
