# Supplementary material for: Anaplastic Lymphoma Kinase Acts in the Drosophila Mushroom Body to Negatively Regulate Sleep
Source: PLoS Genet. 2015 Nov 4;11(11):e1005611. doi: 10.1371/journal.pgen.1005611 (PMC4633181; doi:10.1371/journal.pgen.1005611)
Supplement: S1 Text — (DOCX) [file pgen.1005611.s010.docx]

**Negative geotaxis**

The negative geotaxis assay was adapted from (Barone MC and Bohmann D 2013). *iso31*, *Alk^ts^* and *Alk^ts/1^* flies were raised and kept at 18°C for 7days in a 12h:12h LD cycle after eclosion. The environmental temperature was then raised to 29°C degrees. After 1 d at 29°C, flies were taken out to room temperature 10 min before the tests began. The time between when they were taken out of the 29°C incubator and the assay was finished was limited to 1 hour. 10 flies were transferred, without anesthesia, from a food vial into an empty vial (Fisher Scientific) and then a second empty vial was inverted over, and taped to, the first vial. Each assay consisted of 4 vials of different genotypes. After flies were vigorously tapped down on the bench, they were video-recorded as they climbed up the vial for 10s. The test was repeated 10 times at 1 min intervals for each vial and 5 vials were tested for each genotype. % climbing ability is defined as the percentage of flies that crossed the 4 cm line from the bottom of the vial.

**Quantitative real time PCR analysis**

15 fly heads of each genotype were collected on dry ice. Total RNA was extracted with TRIzol (Life Technologies) and stored at −80°C. Reverse transcription was performed with 1 μg of RNA using a High Capacity cDNA Reverse Transcription kit (Applied Biosystems). qPCR reaction was performed in triplicate with SYBR green (Life Technologies) using the following primers: AlkF: 5’ CGA ACA CCT ATG CTC CTC ACA C 3’, AlkR: 5’ GAA AAC CAT CCG AAT AGT TGC C 3’.
