# Supplementary material for: Anaplastic Lymphoma Kinase Acts in the Drosophila Mushroom Body to Negatively Regulate Sleep
Source: PLoS Genet. 2015 Nov 4;11(11):e1005611. doi: 10.1371/journal.pgen.1005611 (PMC4633181; doi:10.1371/journal.pgen.1005611)
Supplement: S1 Table — (DOCX) [file pgen.1005611.s011.docx]

| Table S1. GAL4 expression patterns | |
| --- | --- |
| c309 | MB all lobes, OL, PI, EB, SOG |
| Pdf | LNv clock neurons |
| 17D | MB αβ core |
| C687 | PI, FB |
| C507 | EB |
| D52H | MB all lobes, weaker in female |
| c772 | MB all lobes, OL |
| MB247 | MB αβ, MB γ, PI |
| R71G10 | MB all lobes, PI |
| kurs45 | PI, |
| c107 | EB |
| c232 | EB |
| c584 | PI, FB |
| Dilp2 | PI |
| InSITE106 | PI |
| 201Y | MB αβ, MB γ, PI |
| 104Y | FB |
| mai301 | PI |
| TH | dopaminergic |
| 50Y | PI |
| 36Y | PI |
| c739 | MB αβ, AL, EB, FB |
| DopR1 | weak MB, EB, FB |
| c929 | peptidergic, PI |
| NP1004 | OB, FB, SOG |
| c767 | PI |
| Tdc | octopaminergic, tyraminergic |
| c819 | EB, SOG |
| NP2024 | PI, scattered cells |
| 53b | MB αβ, AL, PI |
| c305a | MB α'β', AL, OL, PI, EB |
| Ddc | dopaminergic and serotoninergic |
| kurs58 | PI |
| R76D11 | MB αβ, MB γ |
| H24 | MB γ, AL, OL, EB, FB |
| 386Y | peptidergic, MB all lobes, PI, OL |
| 7Y | MB αβ,MB γ, OL, EB, FB |
| OK107 | MB all lobes, AL, OL, PI, EB |
| 6B | EB, FB, OL, PI |
| c453 | PI, FB |
| MJ63 | PI, scattered cells |
| 1471 | MB γ, AL, OL, PI, SOG, Glia |
| 30Y | MB all lobes, AL, OL, PI |
| 121Y | MB αβ, MB γ, PI, EB |
| C320 | MB all lobes, AL, OL, PI, EB, FB |
| 238Y | MB all lobes, AL, OL, PI, EB |
| NP1131 | MB α'β', MB γ, OL, PI, EB |
| Cha | cholinergic, MB all lobes, |
|  |  |
| Abbreviations | |
| MB | mushroom body |
| PI | pars intercerebralis |
| OB | optic lobe |
| EB | ellipsoid body |
| FB | fan-shaped body |
| SOG | subesophageal ganglion |
| AL | antennal lobe |
